# Impact, lessons learned and implications from the national dissemination of evidence-based psychotherapies: Informing research and future D&I directions

**DOI:** 10.1186/1748-5908-10-S1-A77

**Published:** 2015-08-20

**Authors:** Bradley E Karlin, Susan D Raffa

**Affiliations:** 1 Education Development Center, Inc., New York, NY 10014, USA; 2 Department of Mental Health, Bloomberg School of Public Health, Johns Hopkins University, Baltimore, MD 21205, USA; 3 Mental Health Services, U.S. Department of Veterans Affairs Central Office, Washington, DC 20420, USA; 4 National Center for Health Promotion and Disease Prevention, U.S. Department of Veterans Affairs, Durham, NC 27705, USA

## 

Relative to their efficacy and recommendation in practice guidelines, evidence-based psychotherapies (EBPs) are delivered at strikingly low rates. Various attempts to promote the delivery of EBPs, generally involving unidimensional dissemination approaches, have yielded limited success. To bridge science and practice in this area, the U.S. Department of Veterans Affairs (VA) has implemented the largest dissemination of EBPs to date (Karlin & Cross, 2014). Based on dissemination science, this initiative has been guided by a national, multi-level model, involving policy, provider, local systems, patient, and accountability levels, for promoting EBP dissemination, implementation, and sustainability. To date, 15 EBPs have been disseminated, and more than 8,000 staff have been trained in one or more of these therapies. Most significantly, the implementation of EBPs in routine clinical settings has led to unprecedented outcomes for a large number of Veterans, with overall effect sizes on multiple process and outcome domains comparable to those reported in randomized controlled trials. This experience provides compelling evidence that the broad translation of EBPs from science to practice is feasible and yields outcomes that are similar to those achieved in highly controlled settings. The current presentation will: (1) review the national, multi-level D&I model and specific strategies for promoting the national dissemination, implementation, and sustainability of EBPs; (2) examine therapist training, patient outcome, and additional data on the effectiveness of this applied D&I initiative based on integrated, national program evaluation data now available on thousands of therapists and Veterans; and (3) identify key lessons learned and significant implications for future research and large-scale dissemination and implementation based on more than 7 years of experience and accumulated data associated with this national initiative. This national "experiment" provides a unique opportunity for real-world D&I, on a broad scale, to inform research and to identify needs and priorities of policymakers and those on the front lines.
